# Editorial for Advanced Antennas for Wireless Communication Systems

**DOI:** 10.3390/mi15010115

**Published:** 2024-01-10

**Authors:** Trushit Upadhyaya, Hari Shankar Singh

**Affiliations:** 1 Department of Electronics and Communication Engineering, Chandubhai S. Patel Institute of Technology, Charotar University of Science and Technology (CHARUSAT), Changa 388421, India; 2 Department of Electronics and Communication Engineering, Thapar Institute of Engineering and Technology, Patiala 147004, India; harishankar.singh@thapar.edu; 3 Centre of Excellence in Emerging Materials (CEEMS), Thapar Institute of Engineering and Technology, Patiala 147004, India

In recent years, there has been a significant expansion in wireless communication, evolving into a global network connecting billions of entities, including individuals and enterprises. This transformation has given rise to intelligent interconnections among various physical objects, such as vehicles, smartphones, habitats, and their occupants. A pivotal technology for the next generation of communication is the Internet of Things (IoT), incorporating cost-effective data collection and dissemination devices like sensors and RFID tags. These facilitate rapid communication between objects and individuals across any location and time, finding applications in remote healthcare, wearables, autonomous vehicles, wireless robots, and smart homes [1,2,3].

As the demand for application-oriented end-user apps continues to grow, the fifth generation of communication (5G) has replaced the fourth generation, requiring a thorough investigation of elements enhancing its applicability. This includes a discussion on antenna design and measures to improve antenna performance [4,5]. Notably, the implementation of 5G in the IoT and the emergence of the sixth generation (6G) have become areas of active research, with 6G expected to be implemented by 2030. The paper aims to cover the evolution from 5G to 6G, emphasizing the integration of IoT with both generations and addressing antenna design and performance enhancement for 5G [6,7,8].

The objective of this Special Issue is to present creative academic papers focusing on antennas for various communication system applications. As high-speed internet connections serve as vital conduits connecting multiple devices, user demands are escalating rapidly due to technological advancements in wireless communication systems. Numerous studies have concentrated on improving microwave and radio frequency (RF) components, especially antenna systems, considered the foundation of wireless systems. Advanced communication antenna technology has significantly progressed in recent years, enhancing communication quality in home and business applications.

Antenna design faces challenges in balancing cost-performance trade-offs and physical parameters while achieving ideal radiation performance. Research is exploring innovative technologies such as terahertz antennas, Massive-MIMO, UWB-MIMO, and MIMO to meet data rate requirements while keeping costs low. The Special Issue seeks designs with optimal performance.

Low-Profile Antenna Design (Contributions 1 and 5): In this work, a low-profile millimeter-wave broadband metasurface-based antenna is used. The characteristic mode analysis (CMA) is utilized in the design and optimization of the suggested antenna to direct the mode excitation. A small, printed circuit board with four sets of differently sized coplanar patches was used to create four neighboring broadside modes that are fed directly by a coaxial probe. Next, by etching slots on the parasite patch, a new resonant mode is produced to increase the low-frequency bandwidth. The radiation performance of the old modes remains mostly unchanged despite the addition of a new mode. Additionally, the orthogonal modes of the selected modes were moved out of the operational region by creating dual slots on the mid-patch fed by the coaxial probe. It lowers the degree of cross-polarization by shifting the selected modes’ orthogonal modes outside of the working band. Further, in Contribution 5, a dual-band printable monopole antenna is created and displayed. With enhanced bandwidth and gain, the proposed antenna responds in a promising manner. With a strength of 3.7 dBi and 5.26 dBi, the antenna may radiate from 3.49 GHz to 3.82 GHz and from 4.83 GHz to 5.08 GHz, respectively, with a bandwidth of 9.09% and 5.06%. The newly created antenna is novel in that it has enough engineered resonating elements without requiring an additional reactive component. The substrate is a reasonably priced FR-4 laminate. For both resonances, this structure has an efficiency of more than 83%.

Moreover, Ultrabroadband Antenna Design (Contribution 2): The requirement for fast communication has made it possible to construct THz antennas with high data rates, speeds, and frequencies. This manuscript describes the design of a THz MIMO antenna with a metamaterial. The suggested two-port antenna design makes use of a split-ring resonator patch that is complementary. To demonstrate the improvement, the design outcomes are also contrasted with a straightforward patch antenna. A 50 dB improvement in isolation is shown in the design. It is possible to attain a broadband width of 8.3 THz using this complimentary split-ring resonator architecture. 90% of the bandwidth is utilized, indicating an ultrabroadband response.

In Contribution 10: For sub-6 GHz MIMO communication, a four-port dielectric resonator (DR)-based multiple-input multiple-output (MIMO) antenna is described. Dual-band resonance was achieved via an aperture feeding the dielectric resonator. The DRA operates at 3.3 GHz and 3.9 GHz in the modes *TE*_01δ_ and *TE*_10δ_, respectively. Without the need for an additional isolation mechanism, the built antenna provides port isolation of greater than 20 dB at the desired frequencies. The simulation calculation was performed using full-wave high-frequency simulation software. At 3.3 GHz and 3.9 GHz, respectively, the antenna’s maximal gain and efficiency are 5.8 dBi and 6.2 dBi, and 88.6% and 90.2%, respectively. There are good MIMO diversity parameters for the suggested resonator.

In conclusion, a range of antennas has been developed within this Special Issue, showcasing their potential to become key candidates for revolutionizing the next generation of wireless communication.
